# Public Awareness of Consumer Products Containing Radioactive Materials: Empirical Evidence from Malaysia

**DOI:** 10.3390/ijerph19042326

**Published:** 2022-02-17

**Authors:** Zuraidah Sulaiman, Hanis Syuhada Ahmad Sugiran, Nornajihah Nadia Hasbullah, Adaviah Mas’od, Suhairul Hashim, David Andrew Bradley

**Affiliations:** 1 Azman Hashim International Business School, Universiti Teknologi Malaysia, Johor Bahru 81310, Johor, Malaysia; hnissyuhada@gmail.com (H.S.A.S.); adaviah@utm.my (A.M.); 2 Faculty of Business and Management, UiTM Melaka, Kampus Bandaraya Melaka, Melaka 75350, Melaka, Malaysia; najihahnadia@uitm.edu.my; 3 Department of Physics, Faculty of Science, Universiti Teknologi Malaysia, Johor Bahru 81310, Johor, Malaysia; suhairul@utm.my; 4 Centre for Applied Physics and Radiation Technologies, School of Engineering and Technology, Sunway University, Bandar Sunway 47500, Selangor, Malaysia; d.a.bradley@surrey.ac.uk; 5 Department of Physics, University of Surrey, Guilford GU2 7XH, UK

**Keywords:** regulatory focus theory, radiation safety knowledge, product knowledge, risk perception, information seeking, purchase intention, protective action decision model, heuristic-systematic model, Malaysia consumers

## Abstract

The emergence of online purchase platforms makes products containing radioactive materials more accessible to consumers. These products are gaining popularity and are widely available and easily accessible in the market today. This study examined how consumer’s psychological factors affect their decision of purchasing products containing radioactive materials in the market. Based on the protective action decision model (PADM) and the heuristic-systematic model (HSM), this study proposed a model to add to the literature on consumer awareness of risky products. In particular, this study investigated which type of regulatory focus message (promotion-focused advertisement or prevention-focused advertisement) is significant in moderating the effects of radiation safety knowledge and product knowledge on risk perception when purchasing products containing radioactive materials. The relationship between consumers’ risk perception and information seeking, which leads to the purchase intention of such products was also investigated. Advertisements with varying regulatory focus messages were randomly distributed to participants to determine whether consumers are more influenced by promotion-focused advertisement or prevention-focused advertisement to mitigate the risk of purchasing products containing radioactive materials. The results revealed that promotion-focused advertising messages evoked a positive effect on consumers’ radiation safety knowledge and product knowledge toward risk perception. However, prevention-focused regulatory advertising messages did not moderate the relationships between both radiation safety knowledge and product knowledge on consumers’ risk perception. This study offers guidelines for manufacturers, sellers, and marketers of products containing radioactive materials, and, importantly, for the government to devise strategies in designing effective social marketing advertisement for business, environmental and societal benefits.

## 1. Introduction

Radioactivity is part of the earth and is present in housing materials, food, and gases (including the air humans breathe), while radioactive elements (i.e., potassium 40, carbon 14, radium 226) exist in human blood and bones [1]. Some consumer products, including household items, also contain radioactive materials [2]. In the health industry, the use of radioactive substances is emerging in medical treatments, including the diagnostic procedures that provide important information for injury, pregnancy, and medical checkup. It is critical to protect oneself from needless radiation exposure, since excessive radiation exposure might pose detrimental health consequences. Meanwhile, numerous consumer products offering health benefits are currently available. These products such as scalar pendant, tourmaline mat, tourmaline magnetic sock, ion energy saver card, energy comb, and volcanic mask are supposed to contain radioactive materials. However, these products can be harmful to consumers when they are continuously in direct contact through consumption.

In Malaysia, a diverse range of consumer products containing radioactive substances are available in the public domain. Users are generally unaware of the presence of radioactivity in these products, as well as the potential harm that radiation exposure can cause under certain conditions. As a result, it is critical to ensure that consumer products contain as little radioactivity as possible. Furthermore, proper guidelines in designing effective marketing advertisement by the government, manufacturers, sellers, and marketers of products containing radioactive materials should be produced to ensure that all vital information on consumer products containing radioactive substances and their potential risks to the environment and society are communicated effectively to the public. The use of radiation in products is becoming more common, which means that more safety rules and practises need to be in place [3]. In alignment with that, the Malaysian government needs to adopt some actions to increase radiation safety and security through the certification of radiation safety. Furthermore, consumer product standard specifications should be developed. This ensures that, even if these products are mishandled and treated as household waste, radiation doses to individual members of the public will be small, thus, posing a negligible radiation risk. Under these conditions, no special regulatory controls would be necessary to protect the public from unnecessary radiation exposure caused by consumer products. As a result, many countries have enacted standards and regulated protection measures, generally with control via exemption levels for radioactive materials in consumer products. Transnational shipment and use of such consumer products may continue to be a serious global issue in the absence of harmonised control.

The passage of the Atomic Energy Licensing Act (Act 304), followed by the establishment of the Atomic Energy Licensing Board (AELB) in 1984, are significant steps that have been taken by the Malaysian government to control, safeguard, and monitor ionising radiation operations in Malaysia. The AELB should assess and approve the products throughout the manufacturing process before they are made available to the public. Most countries, e.g., the USA [4], apply the 1 μSv per year upper limit, while Malaysia follows the LEM-TEK-69 regulation guideline of its Atomic Energy Licencing Board [5], with an effective dose exemption limit of less than 10 μSv per year per product. By executing Act 304, it licenses all activities linked to nuclear technology applications in all sectors. Furthermore, AELB is a national authority holding an important role in promoting the safe and peaceful applications of atomic energy in Malaysia. AELB’s vision is to remain as a relevant authority with credibility in radiation and nuclear safety, security and safeguarding its peaceful uses for sustainable nation development. Nonetheless, there is still a lack of consistency about the regulation; it is necessary for the regulatory bodies to harmonize current acts or to enact new regulations and legislation for the industry [6]. Altogether, this effort will lead to satisfying the consumers’ demand, as well as to meet the current and future needs of sustainability in radiation safety and security systems. Nevertheless, the growing trend of online purchasing activities has eased consumers’ access to radioactive consumer products. Equipped with limited knowledge on the radioactive products, consumers may unconsciously expose themselves to varying amounts of radiation in large doses, causing tissue damage and possible death. Prior studies have asserted that it is crucial to sensitize the general public to the danger of radiation exposure [7,8,9]. There is also research on consumers’ acceptance of irradiated food [10]. A recent study [11] demonstrated Malaysians’, particularly those of Generation Y’s, perception of the severity of potential risks in purchasing local skincare products. Moreover, several studies [12,13,14] have examined the presence of heavy metals in cosmetic products that produce adverse effects to humans. However, there is no published research to date that has observed the empirical evidence of consumers’ behaviour in using consumer products containing radioactive materials, as well as its potential moderating role of advertising framing messages in the consumers’ decision-making process of such products. In line with the development of technology, the consumer products containing radioactive materials are currently produced not only for personal use, but are rather widely available for domestic and international businesses. Despite that there are a number of legal provisions in Malaysia [11], there are still some issues regarding the sale and production of products containing radioactive materials, most of which have claimed to offer health benefits. Some researchers [15] have elaborated that consumers’ safety behaviour is significantly affected by their understanding of clear and precise product risk information. Therefore, clear and convincing advertising messages should be designed to educate individuals on the risk of radiation exposure.

To address this issue, the integration of a comprehensive model is needed in order to discuss consumers’ behavioral response towards radioactive consumer products. In this study, the heuristic-systematic model (HSM) was integrated into the protective action decision model (PADM) to guide the conceptual framework in exploring the purchase intention of products containing radioactive materials. The implementation of only PADM does not allow one to consider information processing that may affect consumers’ risk responses [16,17], while HSM is a potential and valuable research paradigm used in risk information seeking and processing [18,19,20]. Prior studies, such as those by [19,21,22,23] have been applying these theories on the subject of online consumer behavior, acceptance of genetically modified food, anti-nuclear behavioural response, risk perception, air pollution, green consumption and vaccine acceptance. Therefore, the integration of PADM and HSM should provide a comprehensive model to discuss consumers’ behavioral response. The present study asserts that radioactive safety knowledge, product knowledge and risk perception, would further trigger information seeking and information processing. As a result, purchase intention of products containing radioactive material are stimulated. The present study involves the designing of the advertisement stimuli and experiment studies in order to examine whether advertising framing messages using different regulatory focus (promotion focused versus prevention focused) would affect the consumers’ decision-making process and purchase intention of products containing radioactive materials.

## 2. Literature Review and Theoretical Foundation

This section introduces theories that have been utilised in the present study to predict consumers’ decision-making process and to unfold the hypotheses deducted from the PADM, HSM, ABC Model of attitude, and regulatory focus theory on consumers’ purchase intention towards products containing radioactive materials.

### 2.1. Protective Action Decision Model (PADM)

The protective action decision model (PADM) is used to explain responses of an individual towards threatening events. Prior studies have recently used the extended PADM to predict individuals’ behaviours in various risk situations, such as product a recall crisis in the automobile sector [24], environmental hazards and pollution smog-ridden cities [23], and cumulative disaster exposure [25]. The PADM uncovers three types of respondents’ perceptions: stakeholder perception, protective action perception, and threat perception [25]. Radioactive products pose serious environmental concerns, posing health risks to both present and future generations [26]. Thus, the PADM is pertinent to explaining the mitigation of purchasing decisions for products containing NORM.

### 2.2. Heuristic-Systematic Model (HSM)

The PADM, however, does not evaluate the impact of information processing on people’s risk judgments and behaviour development [17]. The heuristic-systematic model (HSM), on the other hand, includes two strategies of a person to process the information: heuristic (superficial) and systematic (effortful). An individual who effortlessly makes a decision and judgment on a matter and merely agrees with the experts, employs the heuristic processing strategies [20]. Others who spend time to ponder, observe, and make comparisons while processing the information, use the systematic processing strategies [23]. Trumbo [27] asserted that the changes in an individual’s attitude depended on the information processing strategies used in considering a matter. Therefore, the HSM can be used to explain how consumers are processing information before making their purchase decisions.

### 2.3. Consumer Attitude Model

The ABC model of attitude consists of affective, behavioural and cognitive elements [28]. The term ‘*affect*’ refers to an individual’s thoughts about an attitude object, ‘*cognitive*’ to an individual’s beliefs about an attitude object, and ‘*behaviour*’ to an individual’s response to an attitude object. In practice, this ABC model implies that the consumers’ feelings and beliefs toward a product directly influence their behaviours or responses. The behavioural dispositions concepts, which include social attitudes and personality traits, predict and explain human behaviour [29] and result in varying levels of commitment to an attitude, depending on the type of attitudes formed by the consumers [30]. The types of attitudes encompass: (1) compliance (attitudes are formed to obtain rewards or avoid punishments from others; this is considered the lowest level of involvement); (2) identification (attitudes are formed to ensure that expectations are met); and (3) internalisation (attitudes are integrated with a personal value system and are considered as the highest level of involvement).

Several attitude theories can be used to explain consumer attitude. Firstly, Katz’s [31] functional theory of attitudes claims that the existence of attitudes depends on the presence of motives. An attitude can provide many functions to a person, but the dominant one will be taken into account in the majority of situations. Thus, the consumers’ attitudes can be tackled by identifying the dominant motives. However, a consumer is prone to encounter inconsistencies between attitudes and behaviours. According to Festiger’s [32] cognitive dissonance theory, the contradictions or dissonance can be resolved by altering one’s attitude or behaviour. The strategies include assisting consumers in making buying decisions, since consumers tend to evaluate products after they have purchased them. This concept is different when consumers possess prior knowledge of a product, but are trying to assimilate it with new information, as explained in Sherif and Hovland’s [33] social judgment theory. A consumer’s initial attitude serves as a frame of reference, and the consumer categorises new information according to the level of acceptance or rejection. Therefore, these theories can be a guideline in explaining consumer’s attitudes toward products with radioactive content.

### 2.4. Radiation Safety Knowledge

Radioactive knowledge refers to an individual’s cognition of products containing radioactive elements, without limiting the element benefits or risks in everyday life. Radioactive materials play significant roles in society, including healthcare, agriculture, energy, and other scientific and technological domains. In healthcare, radiation is a critical tool in the treatment of certain cancer types. While its benefits may be life-saving, large amounts of radiation exposure may result in death [1]. Inadequate knowledge of and exposure to the radiation information may result in pessimistic awareness, as well as fear [2] or, in the worst-case scenario, unintentional exposure to high doses of radioactive materials without protective measures. A vivid understanding of radioactive information and subsequent provision of information on radiation safety may result in a high risk perception, driving individuals to undertake protective actions [15]. The previous study by Hanifah et al. [3] showed a general low awareness among Malaysians about radioactive content and the potential for harm in the circumstances of daily exposure to such media. Through the prolonged use of radioactive products, consumers’ risk of cancer and other serious diseases increases. During the manufacturing stage, it is suggested that the products should be subjected to regulatory inspection and certification that is crucial in reducing radiation exposure. Thus, the hypothesis below is proposed:

**Hypothesis** **1** **(H1).** *Consumers’ radiation safety knowledge positively affects their risk perception of products containing radioactive materials.*

### 2.5. Product Knowledge

Product knowledge refers to the specific information regarding a product, such as the ingredients, safety measures, radiation facts, and any pertinent labels affecting the consumers’ awareness [23]. Such knowledge enables individuals to gain a better grasp of the products, which may result in a high level of risk perception of them. Protective measures give additional information, which enables individuals to take prompt actions to respond to risks [34]. Wei et al. [20] highlighted that consumers’ knowledge on certain products influences their risk perception or judgment on the products and, therefore, inspires them to seek for more information. Furthermore, Wu et al. [34] emphasized that consumers are willing to take protective actions once they are equipped with substantial knowledge on certain products; hence, enabling them to have high levels of risk perception. When risks are recognised, consumers tend to actively seek additional information to evaluate the risks correctly. This process motivates great discernment for consumers in making purchase decisions for products containing radioactive materials. Perhaps, in the related study by Kubota [35], the author mentioned that the Japanese government has conducted regular tests for radioactive material to convince the Malaysian consumers about the status of Hokkaido dairy products. Thus, the following hypothesis is advanced:

**Hypothesis** **2** **(H2).** *Consumers’ product knowledge positively affects their risk perception of products containing radioactive materials.*

### 2.6. Risk Perception

Risk perception refers to an individual’s subjective judgment of the threat posed by the likelihood of a hazard [23]. In this research context, the risk perception represents unnecessary radiation exposure in which consumers should be encouraged to seek pertinent information in order to make an informed purchasing decision [24]. Risk perception constructs were based on previous studies [23] and are part of the PADM [36]. Risk perception usually originates from prior experience; therefore, the initial product knowledge and radioactive safety knowledge are included to predict consumers’ behavioural response [37]. When consumers perceive a product as posing a severe risk, they are more inclined to assess the risks after gathering a wealth of information. Similarly, risk perception considerably affects people’s level of concern, as well as their risk-information-seeking behaviour [38]. Thus, the hypothesis below was proposed:

**Hypothesis** **3** **(H3).** *Consumers’ risk perception positively affects their information seeking on products containing radioactive materials.*

### 2.7. Information Seeking towards Purchase Intention

Uncertainty happens when a void exists in a topic, or when issues arise. Available information in handling issues is frequently insufficient in terms of certainty, the immediacy of the threat, and the necessary safety precautions [39]. Information on how to safely avoid risks is vital to protect individuals from those risks. Prior research suggested that providing customers with knowledge on how to manage risks will improve their risk perception, depending on the issue of the hazards [15]. It can be seen that consumers tend to gather a wealth of information before making a purchase [23]. Hence, this study hypothesises:

**Hypothesis** **4** **(H4).** *Consumers’ information seeking positively affects their purchase intention of products containing radioactive materials.*

### 2.8. Regulatory Focuses on Advertising Messages

The regulatory focus theory identifies the varied levels of individuals’ goal-pursuit based on how they understand information via one of the two modes: promotion-focused and prevention-focused [40]. The development of advertisement messages attuned to the individual psychological constructions depends on how individuals process information; therefore, it is crucial to address social norms in terms of message framing, whether positive or negative [41]. Promotion-focused messages tend to emphasise on gaining positive outcomes, attaining achievements, and aspiring towards an ideal self. In contrast, prevention-focused messages underline the need of achieving safety and security, and avoiding undesirable states of one’s self [42]. Therefore, the hypotheses below were advanced:

**Hypothesis** **5** **(H5).** *When consumers are exposed to a regulatory focus advertising message (either promotion-focused or prevention-focused), there will be a more positive effect of consumers’ radiation safety knowledge on their risk perception of products containing radioactive materials.*

**Hypothesis** **6** **(H6).** *When consumers are exposed to a regulatory focus advertising message (either promotion-focused or prevention-focused), there will be a more positive effect of consumers’ product knowledge on their risk perception of products containing radioactive material.*

### 2.9. Extended Research Model

**Figure 1:**
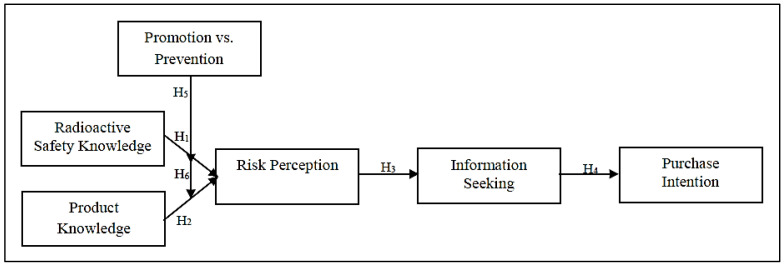
Conceptual Framework.

Drawing from the aforementioned literature, the conceptual framework in the present study was developed and guided by the PADM, the HSM, the ABC model of attitude, and the regulatory focus theory to examine the consumers’ decision-making process when consumers are encountered with consumer products containing radioactive materials. The existence of regulatory focus message as moderating impact that consists of either promotion-focused or prevention-focused message advertisement was also analysed to examine the purchase intention of consumer products containing radioactive materials, as depicted in Figure 1.

## 3. Research Methodology

### 3.1. Study Design

This study used an experimental design with three conditions (promotion-focused stimulus, prevention-focused stimulus, and no stimulus). Through an online survey across Malaysia, 1405 participants were randomly assigned to one of the three conditions. The majority of respondents were female and were from the Malay ethnic, and almost half of the respondents were young, aged between 19 and 24 years old. Most of the respondents had a bachelor’s degree and resided in Johor, a state in the south of Malaysia. Most respondents were single and received a monthly household income of MYR4000 or less.

### 3.2. Stimulus Materials

Two versions of a single-page advertisement were created to establish the regulatory-focused framing. The advertisement copy was adapted from the Radiation in Everyday Life Safety and Security Factsheets (2016) of the International Atomic Energy Agency (IAEA) [1]. The regulatory focus of the body messages was adapted from past research to imply advertisements that seek to promote and prevent radiation exposure in daily life [41,43]. Prior research accentuated the foundation of regulatory focus as a distinction between promotion and prevention, which is exhibited by four distinct modes for pursuing distinct aims. These modes consist of needs, standard target, strategic tendencies, and outcome, as shown in Table 1 [43].

**Figure 2:**
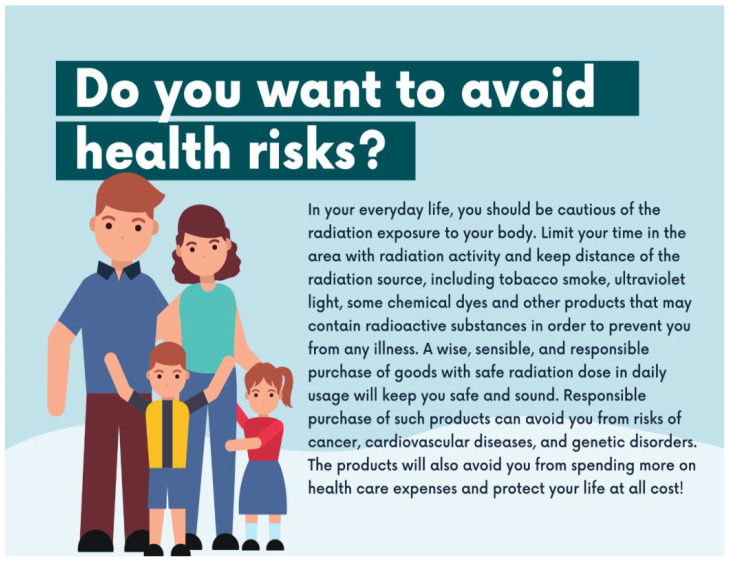
Promotion-Focused Advertisement Stimulus.

**Figure 3:**
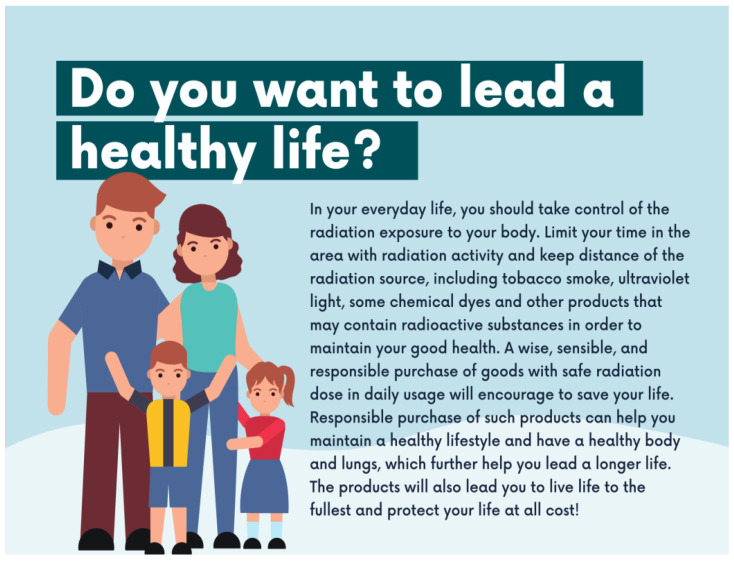
Prevention-Focused Advertisement Stimulus.

Figure 2 illustrates the manipulation of regulatory-focus messaging for promotion-focused advertisement on achieving a healthy life, whereas Figure 3 illustrates the prevention-focused advertising message on avoiding health risks. Both of these regulatory-focus manipulations are set as *generic advertisement stimuli* for the study.

### 3.3. Procedures

In this study, questionnaires were distributed to participants via an online survey platform (i.e., Survey Monkey). Participants were randomly assigned to one of three conditions (promotion-focused stimulus, prevention-focused stimulus, or no stimulus). The questionnaire for this study is divided into three sections. Section A acknowledges the participants of the data collection, and collects their demographic information, such as gender, age, education level, nationality, ethnicity, state of residence, postcode, marital status, employment sector, and income level. Section B serves as a priming stimulus, exposing respondents to a sample of 20 items. Participants were asked simple questions on their previous experiences with products containing radioactive materials in terms of purchase or usage, as well as their knowledge about products containing radioactive materials. While Section B (1) includes questions regarding radiation safety knowledge and product knowledge, as well as the attachment of the *generic advertisement* and *product specific advertisement stimuli*. Finally, Section C presents items of other measurement constructs, which are Risk Perception, Information Seeking, and Purchase Intention. In order to gather the relevant responses to the varying conditions, the respondents were assigned into three different groups; Group A (given *promotion focus stimuli*), Group B (given *prevention focus stimuli*), and Group C (given *no stimuli*). All of the questionnaire items remained unchanged across all groups.

Three sets of questionnaires with one varying condition each were distributed randomly to the participants. Group A respondents received the first questionnaire set with *promotion-focused generic advertisement* and *product specific advertisement* stimuli. Group B received the second set of questionnaires with *the prevention-focused generic advertisement* and *product specific advertisement* stimuli, Group C received the third questionnaire set without any advertisement stimulus. The third group was the control group, whereas the first two, Groups A and B, represented the experimental groups. After completing Section A, participants in the experimental groups were exposed to either a promotion-focused or a prevention-focused advertisement in Section B (1) for as long as they desired, similar to a real-world context. Then, they progressed to Section C, completing the measurement instrument. Finally, participants were debriefed and thanked for their participation and commitment in this study.

### 3.4. Measures

All items used in the measures were adopted from prior studies on risky product purchases and rated a five-point Likert-scale (1 = strongly disagree to 5 = strongly agree), as shown in Table 2.

### 3.5. Statistical Techniques

To accomplish the study objectives, data were analysed using the statistical software SEM-PLS (3.2.8). The software was used to extract the multivariate qualities of the data, which facilitates the analysis of the measurement and structural models.

## 4. Results

### 4.1. Demographic Profile

Out of 1405 participants, only 1065 responses (75.8% response rate) were usable for analysis. Among 1065 respondents, Table 3 summarises the respondents’ profiles.

The majority of respondents (*n* = 738; 64%) were female; almost half were between the ages of 19 and 24 (*n* = 444; 41.7%). Meanwhile, more than half of respondents had a bachelor’s degree (*n* = 665; 62.4%) and were Malay (*n* = 856; 80.4%). The respondents resided in various states in Malaysia, with the majority from Johor (*n* = 340; 31.9%), a state in the south of Malaysia. Most respondents were single (*n* = 648; 60.8%) and received a monthly household income of MYR4000 or less (*n* = 750; 70.4%).

### 4.2. Priming the Respondents

Respondents who had been primed were asked two introductory questions on their prior experience with or use of radioactive materials, as well as their prior knowledge of products containing radioactive materials. A total of twenty items (priming stimuli) were organized in two groups: Items A and Items B. Prior to exposing respondents to the advertisement stimulus, these priming questions were used to engage and elicit memories of respondents’ previous usage and early understanding of products containing radioactive materials.

### 4.3. Manipulation Check

Manipulation check is necessary when treatments indirectly manipulate other constructs. Following the verification of success, manipulation check is used to determine whether an experiment was carried out successfully by altering the experiment conditions. For this study, seventy members of the public were recruited for manipulation check. In order to get an acceptable capacity for detecting fair prevalent issues (0.10), a sample size of at least 30 is sufficient. The pre-testing survey for manipulation check was conducted online. The primary objective of the pre-testing session was to determine the differences in message focus of the advertisements (promotion vs. prevention) in order to raise awareness about the purchase intention of products containing radioactive materials. By clicking on the link, respondents were routed to the informed consent form. They were then instructed to carefully evaluate two distinct regulatory focus message structures that appeared. In this pre-test, respondents were assigned to both of the advertisement stimuli.

Single sample t-test was conducted to compare the consumers’ perception of both advertisement stimuli (*promotion regulatory focus advertising message* and *prevention regulatory focus advertising message*). There was a significant difference in the scores for promotion regulatory focus advertising message (*M* = 3.50, *SD* = 1.28) and prevention regulatory focus advertising message (*M* = 3.00, *SD* = 1.27) conditions; with *t* (69) = 22.83, *p* = 0.00. The results confirmed that consumers perceived both stimuli to be different from one another; hence, manipulation was successful.

### 4.4. Fitness of the Measurement

The assessment of the measurement model quantifies the loading of each measurement item. The validity and reliability of the research instrument was confirmed using average variance extracted (AVE), as well as composite reliability (CR), values. Table 4 summarises the results of the measurement model assessment. 

AVE is a convergent validity indicator that measures the variance captured by a construct in relation to the variance attributable to measurement error [52]. In this study, the AVE values for all variables were greater than the 0.50 acceptable threshold, confirming the measurement validity [53]. Similar to Cronbach’s Alpha, CR (construct reliability) measures the internal consistency of scale items [54], which should be higher than 0.6 to 0.7 [52]. As shown in Table 4, the CR values for all variables in this study were greater than the acceptable threshold, except for risk perception, 0.587, close to 0.6. In short, the results of the measurement model assessment confirmed the reliability and validity of the measurement items and constructs.

### 4.5. Structural Model of Hypotheses Testing

**Figure 4:**
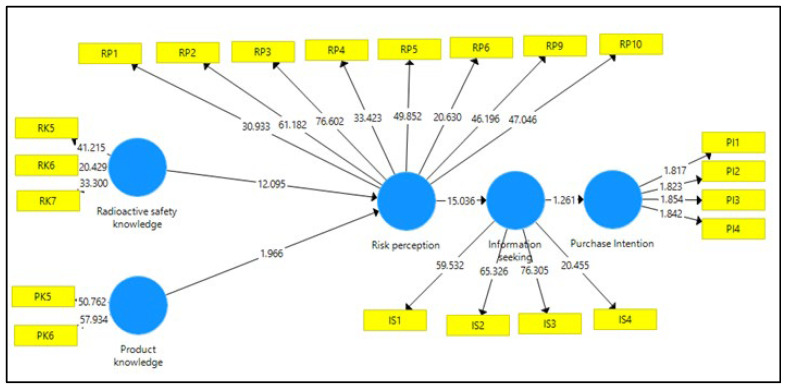
Structural Model Assessment of the Study.

The results of the measurement model assessment are depicted in Figure 4 and the hypotheses testing are summarised in Table 5. All variables showed a positive direct relationship with all values, exceeding the recommended threshold (*p*-value < 0.05, *t*-value > 1.645), except for H_4_. The supported hypotheses, H_1_ and H_2_, indicated that radiation safety knowledge and product knowledge positively affected consumers’ risk perception of products containing radioactive materials. A similar positive relationship supported H_3_, in which risk perception positively affected consumers’ information seeking on products containing radioactive materials. However, for H_4_, information seeking did not positively affect consumers’ purchase intention of products containing radioactive materials.

As mentioned earlier, radiation safety knowledge refers to the individual’s mental cognition or understanding of risks and benefits associated with radioactive elements in life, whereas product knowledge refers to the specific information about particular products, such as ingredients, safety measures, radiation facts, and any pertinent labels regarding the product risks and benefits. The findings of the present study showed that radiation safety and product knowledge positively affected consumers’ risk perception of products containing radioactive materials in Malaysia. This implies that when Malaysian consumers are equipped with radioactive information or sufficiently educated on radioactive knowledge, they are aware of the benefits and risks. Consumers’ awareness of risks may lead to high risk perception, hence, encouraging protective behaviours. Having product knowledge enables customers to gain a better understanding of the products, which may result in a heightened risk perception of products containing radioactive materials.

Risk perception refers to an individual’s subjective judgment of the possibility of a hazard. The findings revealed that risk perception of products containing radioactive materials positively affected consumers’ information seeking for such products. This result suggests that when the risks have been identified, consumers tend to actively seek more information to evaluate the exact risks. In other words, when Malaysian consumers perceive that products propose a potentially severe risk, they are highly likely to assess the risks through equipping themselves with an abundance of information. As a result, this process may either motivate or demotivate consumers to purchase products containing radioactive materials.

Providing consumers with the information on how to safely avoid the radioactive risks is crucial to prevent them from any potential risks. The findings of the present study showed that Malaysian consumers’ information seeking did not positively affect their purchase intention of products containing radioactive materials. It can be inferred that, although Malaysian consumers make attempts to get as much information as possible about products containing radioactive materials, the gathered information may not be sufficient to assist them in making the purchase decisions.

### 4.6. Moderating Effects

The multi group analysis (MGA) approach was applied to examine the moderating effects of regulatory focus message (*either promotion or prevention*) on the relationships between consumers’ radiation safety knowledge on their risk perception of products containing radioactive materials.

H_5_ hypothesised that regulatory focus advertising message (prevention and promotion) would moderate the positive relationship between radiation safety knowledge and risk perception. The results of path analysis, the moderating effects of prevention-focused messaging (*b* = 0.368; *t* = 5.973 **; *p*-value = 0.000) and promotion-focused messaging (*b* = 0.414; *t* = 10.026 **; *p*-value = 0.000) were verified. All values met the recommended threshold (*p*-value < 0.05, *t*-value > 1.645), as summarised in Table 6.

The results revealed that when Malaysian consumers were exposed to promotion-focused advertisement, their radiation safety knowledge positively affected their risk perception of products containing radioactive materials (*b* = 0.414 for the promotion-focused group is higher than *b* = 0.368 for the prevention-focused group). The same goes for when Malaysian consumers were not exposed to any advertisement at all, as in the control group, whereby their radiation safety knowledge resulted in the highest positive effect on their risk perception (*b* = 0.513).

In comparison of all the groups, no advertising message and promotion-focused advertising seemed to hold higher moderating effects on the relationship between Malaysian radiation safety knowledge on their risk perception.

In a similar vein, the moderating effects of regulatory focus advertising messaging (*either promotion-focused or prevention-focused*) on the relationships between consumers’ product knowledge and risk perception of products containing radioactive materials, as hypothesised in H_6_, were investigated. Based on the analysis summarised in Table 7 and the recommended threshold (*p*-value < 0.05, *t*-value > 1.645), prevention advertising messaging did not moderate the relationship between product knowledge and risk perception (*b* = −0.013; *t* = 0.231 **; *p*-value = 0.818). However, promotion-focused advertising messaging moderated the relationship between product knowledge and risk perception, with a value of (*b* = 0.113; *t* = 2.067 **; *p*-value = 0.039). For the control group, the effect of product knowledge remained insignificant to influence risk perception (*b* = 0.134; *t* = 1.951 **; *p*-value = 0.052). These results showed the significance of the stimulus’ moderating effect, i.e., the promotion-focused message on the relationship between consumers’ product knowledge and risk perception of products containing radioactive materials in the experimental groups.

The results revealed that when Malaysian consumers were exposed to advertising messages focused on promotion rather than prevention, their product knowledge more strongly benefited their risk perception of products containing radioactive materials.

## 5. Discussion

Evidently, it is vital for business communication to include additional and detailed information about a product’s benefits to distinguish it from other products. This ensures that manufacturers, sellers, and marketers of products containing radioactive materials, as well as the government, would convey persuasive messages about the potential benefits of radioactive products. The research findings are in line with those of previous research on promotion-focused advertisements [55]. This structure of advertisement has been used to promote various sustainable products and services, including organic food, herbs, natural cosmetics and tourism, technology and infrastructure [56,57,58,59,60].

The findings also suggest that Malaysia must establish standards for approving consumer products containing radioactive materials before they are released to the public for everyday usage. Similarly, manufacturers must adhere to standards to ensure the radiation dose to individual members of the public is as low as reasonably possible. Such convergence and synergy of practice will be a critical step toward designing and producing beauty and healthcare products for public use.

The inclusion of promotion-focused messaging as a moderator is consistent with Tyufekchieva and Reichhart’s [61] assertion that before message receivers could evaluate a message content, their interests must be instantly sparked so that they would feel motivated to read it. Thus, companies need to pay more attention to making the message subject more attractive, in a way that immediately appeals to consumers. An appealing heading motivates readers to read and digest the advertising message [62].

## 6. Conclusions

This study offers guidelines for the manufacturers, sellers, and marketers of products containing radioactive materials and, importantly, for the government to devise strategies in designing effective social marketing advertisements for business, environmental and societal benefits. In promoting hedonic products, the promotion regulatory focus messaging appears to be more effective than prevention focus messaging [63,64,65,66]. In Malaysia, companies and governments should deploy promotion-based regulatory focus messaging in advertisements as an ethical strategy to visibly highlight the positive outcomes of products containing radioactive materials.

## 7. Theoretical and Practical Contributions

This study extends the application of PADM, with the inclusion of regulatory focus advertising messaging as a moderating variable. Although PADM is a comprehensive model, there is a limitation that hinders researchers’ ability to evaluate the impact of information processing related to risk judgments and behaviour development [17]. Therefore, HSM was integrated to overcome the limitation of this model. The HSM includes two strategies for a person to process information: heuristic (superficial) and systematic (effortful). Another theoretical contribution is the expansion of this framework with regulatory focus messaging as the advertising stimuli. The findings showed significant relationships between radiation safety knowledge and both regulatory focus messaging groups (promotion and prevention). In short, it is pivotal for marketers to tailor information related to the products to match with the regulatory focus of consumers in order to produce high-quality arguments that match with consumers’ mindsets [56]. For practical implications, this study offers a better understanding of how consumers actually perceive and evaluate information before making their decision to purchase products containing radioactive materials. Simultaneously, the advertising design structure proposed in this study may be adopted by manufacturers, sellers, and marketers of products containing radioactive materials, and, importantly, by the government to devise strategies in designing effective social marketing advertisements for business, environmental and societal benefits.

## 8. Limitation and Further Research

This study has limitations that provide several directions for future research. First, the study only focused on the general respondents, who did not belong to a specific generation. It is recommended that future studies focus on the younger generation, as it is expected that this demographic segment pays more attention to these kinds of current issues [67]. Second, it is possible for future researchers to explore other factors or theories that were not considered here. Third, it would be interesting to explore other textual or image stimuli advertisements. Future studies can also implement other stimuli that are integrated by technology, such as using videos and hashtag messages to increase the effectiveness of the message delivered.

## Figures and Tables

**Table 1:** Differences between Promotion-Focused and Prevention-Focused Advertisements.

	Promotion-Focused	Prevention-Focused
Needs	Focused on growth and progress	Focused on security and protection
Standard Target	Reflected by hopes and aspiration emphasized on ideal self	Reflected by duties and obligations emphasized on ought self
Strategic Tendencies	Approaching the desired state	Avoiding the non-desired state
Outcome	Presence of positive outcomes	Absence of negative outcomes

**Table 2:** Measurements of Constructs, Items and References.

Author (Years)	Measures (Adopted and Adapted)	Rephrased Scale Items
[44,45]	**Radiation Safety Knowledge**	1. I am familiar with the causes or sources of radiation.
		2. I am familiar with the harm of radiation to human health.
		3. I am familiar with the harm of radiation to my daily life.
		4. I am familiar with the protective measures against radioactive products.
		5. Wearing the products containing radioactive substances frequently may cause high dose of radiation exposure.
		6. Products containing radioactive substances and human skin should be covered by clothing to reduce the risk of radiation exposure.
		7. Increasing the distance from the products containing radioactive substances prevents the high dose radiation exposure.
		8. Products containing radioactive substances should be disposed in the bin when they are no longer in use.
[15,24,34]	**Product Knowledge**	1. I know what kind of products containing radioactive substances.
		2. I know many things about products containing radioactive substances such as energy pendant and volcanic mask.
		3. I am aware there are some products that comply with the radiation safety standards.
		4. I know more about products containing radioactive substances than others around me.
		5. If products comply with the radiation safety standards, they can protect me from the harm of radiation exposure.
		6. If products comply with the radiation safety standards, they can benefit the consumers.
[46,47,48,49]	**Risk Perception**	1. It is dangerous to use products that are containing radioactive substances (such as energy pendant).
		2. I am worried that products containing radiation substances will damage my health.
		3. Continuously wearing products with radioactive substances would seriously damage my health due to beta radiation exposure.
		4. Continuously wearing products with radioactive substances will cause me financial loss due to possible medical expenses.
		5. The risk that I take when I buy products containing radioactive substances is high.
		6. There is high probability that the products containing radioactive substances will not function as per my expectation.
		7. There is high probability that others would think less highly of me when I buy products containing radioactive substances.
		8. It is illegal to buy products containing radioactive substances.
		9. The radioactive product raises the risk of skin disease due to beta radiation exposure.
		10. I am worried that the radiation exposure will damage the health of my loved ones.
		11. I think products containing radioactive materials are still dangerous although they have been approved/endorsed by the authority.
[50,51]	**Information Seeking**	1. I want to search for more information about products containing radioactive substances.
		2. I have to search for more information about products containing radioactive substances.
		3. I am concerned with the latest news of products containing radioactive substances.
		4. I actively search for information about products containing radioactive substances and hope they are available.
[34]	**Purchase Intention**	1. I am willing to purchase products containing radioactive substances to improve my health.
		2. I am thinking about purchasing products containing radioactive substances to improve my health.
		3. I intend to purchase products containing radioactive substances to improve my health.
		4. I think it is quite necessary to purchase products containing radioactive substances to improve my health.
		5. I am the primary decision maker purchasing these products containing radioactive substances.

**Table 3:** Profile of Respondents.

Demographic	Category	Number of Respondents			Percentage (%)
		Set A	Set B	Set C	
Gender	Male	176	104	47	31%
	Female	301	256	181	69%
Age	18 years old and below	2	2	4	0.7%
	19 years old–24 years old	151	154	139	41.7%
	25 years old–40 years old	197	144	69	38.5%
	41 years old–56 years old	113	57	15	17.4%
	57 years old–66 years old	13	3	0	1.5%
	67 years old–75 years old	1	0	1	0.2%
	76 years and above	0	0	0	0%
Highest Education Level	SPM or equivalent	24	19	10	5%
	STPM or equivalent	11	13	2	2.4%
	Diploma or equivalent	59	40	23	11.5%
	Bachelor’s Degree	265	229	171	62.4%
	Master or PhD	113	57	21	17.9%
	Others	5	2	1	0.8%
Nationality	Malaysian	477	360	228	100%
	Non-Malaysian	0	0	0	0%
Ethnicity	Malay	372	306	178	80.4%
	Chinese	69	29	38	12.8%
	Indian	15	10	8	3.1%
	Others	21	15	4	3.7%
State of Residence	Perlis	3	2	1	0.6%
	Penang	19	4	6	2.7%
	Kedah	13	14	7	3.2%
	Perak	16	7	4	2.5%
	Kelantan	12	3	1	1.5%
	Terengganu	15	9	8	3.0%
	Selangor	127	86	58	25.4%
	Pahang	9	14	18	3.8%
	Negeri Sembilan	24	17	10	4.8%
	Melaka	18	16	5	3.7%
	Johor	138	119	83	31.9%
	Sabah	11	12	1	2.3%
	Sarawak	29	13	5	4.4%
	W.P Kuala Lumpur	39	32	17	8.3%
	W.P Putrajaya	4	9	4	1.6%
	W.P Labuan	0	3	0	0.3%
Marital Status	Single	250	212	186	60.8%
	Married with no children	34	28	9	6.7%
	Married with children	190	115	30	31.5%
	Others	3	5	3	1%
Income Level	RM 1000 and less	154	134	83	34.8%
	RM 1001–RM 2500	70	45	79	18.2%
	RM 2501–RM 4000	72	77	36	17.4%
	RM 4001–RM 5500	66	29	8	9.7%
	RM 5501–RM 7000	51	37	14	9.6%
	More than RM 7001	64	38	8	10.3%
Occupation	Architecture and Engineering Occupations	18	20	14	4.88%
	Art, Design, Entertainment, Sports, and Media Occupations	5	1	2	0.75%
	Building and Grounds Cleaning and Maintenance Occupations	0	0	1	0.09%
	Business and Financial Operations Occupations	26	10	11	4.41%
	Community and Social Services Occupations	6	4	0	0.94%
	Computer and Mathematical Occupations	9	6	6	1.97%
	Construction and Extraction Occupations	2	3	2	0.66%
	Education, Training, and Library Occupations	68	38	28	12.58%
	Farming, Fishing, and Forestry Occupations	3	2	3	0.75%
	Food Preparation and Serving Related Occupations	4	2	4	0.94%
	Healthcare Practitioners and Technical Occupations	17	8	2	2.54%
	Healthcare Support Occupations	11	8	3	2.06%
	Installation, Maintenance, and Repair Occupations	8	6	3	1.6%
	Legal Occupations	8	5	2	1.41%
	Life, Physical, and Social Science Occupations	6	14	1	1.97%
	Management Occupations	53	45	11	10.23%
	Military Specific Occupations	0	3	3	0.56%
	Office and Administrative Support Occupations	31	21	9	5.73%
	Personal Care and Service Occupations	1	0	5	0.56%
	Production Occupations	4	14	16	3.19%
	Protective Service Occupations	6	3	1	0.94%
	Sales and Related Occupations	17	7	15	3.66%
	Transportation and Material Moving Occupations	4	3	2	0.85%
	Student	131	117	71	29.95%
	Unemployed	39	20	13	6.76%

**Table 4:** Results Summary for Assessment of the Measurement Model.

Variables	AVE	CR
Product Knowledge	0.897	0.814
Radiation Safety Knowledge	0.819	0.603
Risk Perception	0.908	0.587
Information Seeking	0.888	0.666
Purchase Intention	0.960	0.857

**Table 5:** Hypotheses Results for Direct Effects (H1 to H4).

Hypothesis	Path for Direct Effects	*β* (Path Coefficient)	*t*-Value	*p*-Value	Decision
**H1**	Radiation Safety Knowledge → Risk Perception	0.421	12.095	0.000	Hypothesis Supported
**H2**	Product Knowledge → Risk Perception	0.074	1.966	0.043	Hypothesis Supported
**H3**	Risk Perception → Information Seeking	0.421	15.036	0.000	Hypothesis Supported
**H4**	Information Seeking → Purchase Intention	−0.054	1.261	0.191	Hypothesis Rejected

**Table 6:** Hypotheses results for moderating effects (H5).

Hypothesis	Path for Moderating Effect	β (Path Coefficient)		*t*-Value		*p*-Value		Decision
**H5**	Radiation Safety Knowledge * Regulatory Focus Advertising Message → Risk Perception	Control	0.513	Control	7.402	Control	0.000	Hypothesis Supported
		Prevention	0.368	Prevention	5.973	Prevention	0.000	Hypothesis Supported
		Promotion	0.414	Promotion	10.026	Promotion	0.000	Hypothesis Supported

**Table 7:** Hypotheses results for moderating effects (H6).

Hypothesis	Path for Moderating Effect	β (Path Coefficient)		*t*-Value		*p*-Value		Decision
**H6**	Product Knowledge * Regulatory Focus Advertising Message → Risk Perception	Control	0.134	Control	1.951	Control	0.052	Hypothesis Rejected
		Prevention	−0.013	Prevention	0.231	Prevention	0.818	Hypothesis Rejected
		Promotion	0.113	Promotion	2.067	Promotion	0.039	Hypothesis Supported

## Data Availability

The dataset analysed in this study are available upon request from the authors.
